# Assessment of service readiness for maternity care in primary health centres in rural Nigeria: implications for service improvement

**DOI:** 10.11604/pamj.2021.40.151.25976

**Published:** 2021-11-11

**Authors:** Lorretta Favour Chizomam Ntoimo, Julius Ogungbangbe, Wilson Imongan, Sanni Yaya, Friday Ebhodaghe Okonofua

**Affiliations:** 1 Women´s Health and Action Research Centre, Benin City, Nigeria,; 2 Federal University, Oye-Ekiti, Oye-Ekiti, Ekiti State, Nigeria,; 3 Essential Metadata Analyst, Lagos, Nigeria,; 4 University of Ottawa, Ottawa, Canada,; 5 Centre of Excellence in Reproductive Health Innovation (CERHI), University of Benin, Benin City, Nigeria,; 6 Department of Obstetrics and Gynaecology, University of Benin, University of Benin Teaching Hospital, Benin City, Nigeria

**Keywords:** Primary health centres, physical facilities, medical equipment, personnel, medicines, Nigeria, maternal and child health, service readiness

## Abstract

**Introduction:**

several scientific reports from studies across Nigeria revealed a higher incidence of maternal mortality in rural parts of the country as compared to the urban areas. Part of the reasons is the paucity of health care infrastructure and personnel. This study was designed as part of an intervention program with the goal to improve the access of pregnant women to skilled pregnancy care in rural Nigeria. The specific objective of the study was to determine the nature and readiness of Primary Health Centres (PHCs) in two Local Government Areas (LGAs) in rural parts of Edo State, Southern Nigeria to deliver effective maternal and child health services.

**Methods:**

the study was conducted in 12 randomly selected PHCs in the two LGAs. Data were obtained with a semi-structured questionnaire administered on health workers and through direct observation and verification of the facilities in the PHCs. The results obtained were compared with the national standards established for PHCs in Nigeria by the National Primary Health Care Development Agency (NPHCDA). Descriptive statistics were used to analyze the data.

**Results:**

the results showed severe deficits in buildings and premises, rooms, medical equipment, essential drugs, and personnel. Only 40% of items recommended by the NPHCDA were available for buildings; 41% of the PHCs had facilities available in the labour ward; while less than 30% had the recommended facilities in the antenatal care rooms. Only one PHC had a laboratory space, with only one item (a dipstick for urine analysis) identified in the laboratory. None of the PHCs had ambulances, mobile phones, internet or computers. There was no nurse/midwife in 4 PHCs; only one nurse/midwife each were available in 8 PHCs; while there was no Environmental/Medical Records Officer in any PHC. About 26% of the essential drugs were not available in the PHCs.

**Conclusion:**

we conclude that PHCs in Edo State, Nigeria have severe deficits in infrastructural facilities, equipment, essential drugs and personnel for the delivery of maternal and child health care. Efforts to improve these facilities will help increase the quality of delivery of maternal and child health, and therefore reduce maternal and child mortality in the country.

## Introduction

Available data indicate that Nigeria has one of the highest maternal mortality ratio in the world estimated at 814 per 100,000 live births with an uncertainty interval of 596-1180 [1]. Several scientific reports from studies across the country revealed a higher incidence of maternal mortality in rural parts of the country as compared to the urban areas [2,3]. In a comparison based on an analysis of the Nigeria Demographic Health Survey, the risk of maternal mortality in the rural part of the country was 1 in 32, whereas it was 1 in 45 in the urban areas [2]. Part of the reasons for the higher rate of maternal mortality in rural parts of Nigeria is the paucity of health care infrastructure and personnel to handle complications of pregnancy [4-7]. Whereas up to 50 per cent of Nigerians reside in rural areas [8], less than 10 per cent of the country´s health resources are deployed to rural locations. Most referral hospitals are located in urban areas, and with poor and inadequate transportation, rural residents often have limited opportunities to obtain sophisticated maternity care when needed [6,9].

Nigeria subscribes to the global principles of universal health coverage and health for all [10-12]. These principles stand the chance of reaching the most vulnerable persons and communities around the world with essential information and services to improve maternal health and decreasing maternal and neonatal mortality. As such, over the years, Nigeria has sought to implement primary health care services at both national and sub-national levels as the approach to reach the most vulnerable citizens in the country, especially those in rural areas [13-16]. While this policy has taken root since the onset of the new democratic experience in Nigeria, PHC infrastructure in the country remains weak and poorly funded, with resulting low utilization of available PHCs for maternal and child health care [7,17,18].

Our recent studies showed that less than 47 per cent of pregnant women in a rural part of Edo State where only PHCs are available for maternal and child health care, utilize the facilities for antenatal and delivery care [18]. Through several qualitative studies, we were able to establish that one of the major reasons that women do not use available PHCs for skilled pregnancy and child health care in the rural areas is the perception that PHCs are of low quality, and that qualified staff and equipment may not be available to deliver optimal care [6,19,20]. Especially for the less educated rural women, they perceive the treatment offered in PHCs as not better than those provided by traditional birth attendants (TBAs). This largely accounts for the greater tendency for these categories of women to seek care with TBAs rather than in the PHCs.

Thus, it is clear that if the quality of PHC delivery is not improved in Nigeria, it is unlikely to enable the attainment of universal coverage for maternal and child health care as envisaged by the Federal government. It is within this context that we undertook a quality assessment of selected PHCs in Edo State as part of our comprehensive efforts to identify appropriate interventions for improving the uptake of PHCs for maternal and child health care in the country. We believe that only by addressing the demand and supply factors that limit health care utilization will a comprehensive paradigm be developed for the increasing women´s access to skilled pregnancy care in rural Nigeria.

Several efforts have been devoted to assessing the readiness of PHCs for delivery of effective health care [21-24]. However, such efforts have focused on PHCs overall, rather than those located in rural areas. In 2017, Oyekale [21] reported a national study with a representative sample of 2,480 PHC facilities in the country based on 2013/2014 World Bank health Service Delivery Indicator (SDI) data to assess the preparedness of national PHCs for service delivery. The report focused exclusively on essential drugs and medical equipment and showed evidence that they did not meet the expected benchmarks for effectiveness. However, other indicators of service performance were not reported. Although the data were national in scope, it fell short because it was based on self-reporting of the indicators, and were not supported by site verification. The fact that the health facilities did not provide information on personnel and their capacity also limited the specific usefulness of the study for the design of specific interventions.

Our research was designed from a translational and interventional perspective with the aim to offer specific recommendations to policymakers for improving various components of PHC delivery in order to promote and scale service utilization of PHCs in Nigeria. Thus, extending the past studies, our study examined essential drugs and medical equipment as well as other indicators of service performance as stipulated in the minimum standards for PHCs in Nigeria such as building and premises, sufficient rooms and space, laboratory, separate room for cleaners, staff room, record room, other general equipment such as mobile phone, ambulance, computer, and internet services; and personnel. The specific research question we posed was “what is the nature and readiness of the physical facilities, medical equipment, personnel and essential drugs in selected PHCs in the two LGAs?” We then followed up with an analysis and specific recommendations.

## Methods

**Study design/setting:** this study employed a cross-sectional descriptive design to assess the availability of essential physical facilities, equipment, staff/personnel and essential drugs in the PHCs as part of baseline research for an interventional study on increasing access to skilled pregnancy care in rural Nigeria. The study setting was two predominantly rural Local Government Areas (LGAs), Esan South-East and Etsako East in Edo State, Nigeria. Twelve PHCs were randomly selected from a list of 51 PHCs in the two LGAs. Edo state is one of the 36 states of Nigeria located in the Southern part of the country. The state consists of 18 LGAs with an estimated population of 4 million [25].

**Data collection:** data were collected from July 29 to August 16, 2017 with a questionnaire which was administered through a face-to-face interview with the Nurse/Midwife or Health Attendant in charge of the PHCs using a Computer-Assisted Personal Interview (CAPI). The responses were verified by direct observation. All the facilities, equipment, and drugs that were mentioned during the interview were sighted by the interviewers. The interviews and sighting were conducted by trained data collectors who were knowledgeable about essential medicines, dressing and medical devices. The questionnaire contained three major sections, with the content of each section drawn from the National Primary Health Care Development Agency minimum standard for primary health care in Nigeria [15]. Section 1 contained two sub-sections with 12 items on buildings and premises such as the requirement for a minimum land area of 4.200 square metres, whether the building was painted green, and the availability of a clean water source from a motorized borehole among others. The second sub-section fielded 14 items on availability of sufficient rooms and space to accommodate a waiting/reception area for child care, antenatal care, health education and oral rehydration therapy corner, adolescent health service room, and two consulting rooms among others.

Section 2 contained questions about medical equipment and personnel. This section had 12 sub-sections: 36 items in female ward such as angle poised lamp, artery forceps (medium) and bed pan among others, 23 items in infant and child welfare ward such as basket with lid for ORS, ceiling fan and stainless covered bowl for cotton among others, 53 items in the labour room such as delivery couch, dissecting forceps, dressing trolley and fetal stethoscope among others, 35 items for first stage room such as stainless bedpan, bowls stainless steel with stand, ceiling fan, 25 items in antenatal/interview room such as examination couch, stainless galipot, latex gloves, 29 items in the laboratory such as kidney dish, centrifuge (manual), 15 items in cleaning room such as brooms, mops, 19 items in the consulting cubicle such as examination couch, hammer (reflex), 8 items in staff room such as chair, table, dust bin, 7 items in the record room such as table, plastic chairs, safe, 7 items for other requirements such as ambulance vehicle, computer, communication facility (mobile phone or communication radio) among others, and 12 categories of personnel. Twelve categories of personnel is the standard requirement in a PHC including 1 medical officer, 1 Community Health Officer (CHO), 4 nurses/midwife, 3 Community Health Extension Workers (CHEW), 1 Pharmacy technician, 6 Junior CHEW, 1 Environmental Officer, 1 Medical Records Officer, I Laboratory technician, and supporting staff comprising 2 Health Attendant/Assistant, 2 Security personnel, and 2 General Maintenance Staff. In all, a total of 24 staff/personnel should be in a PHC. Section 3 contained questions on 93 essential drugs which included anaesthetics, analgesics, anti-allergies, anticonvulsants, and antidotes, among others.

Questions were fielded on the availability of the expected items according to the prescribed national standards and the response options were: 1) available (when the items are available and meet the required standards and 2) not available, when the items are not available or do not meet the standards. Where applicable, adequacy was assessed if more than one of a specific item is expected. For instance, the minimum standard for PHCs is two consulting rooms. In this case, we assessed both availability and adequacy. The full content of the questionnaire (site assessment) is available in a public open access repository, OpenICPSR.

**Data analysis:** all the analyses were conducted with Stata 12.0 for windows. To identify the number of items available in the PHCs, the responses were aggregated. A dummy variable was generated for each item, available was coded 1 and not available was coded 0. The least number of available items in each segment is zero whereas the highest is equal to the number of expected equipment or item in the particular segment: 12 for physical facilities, 93 for essential drugs and 24 for staff/personnel among others. A test of significant difference in the availability of the items between the two LGAs was conducted using the Mann-Whitney test. The result was insignificant for all the segments and the probability that Esan South East is greater than Etsako East in the availability of the expected items was also insignificant. Thus, the results are presented without disaggregating by LGA. The results are presented using absolute number, percentage, mean with standard deviation (SD) and median with interquartile range (IQR).

**Ethical approval:** the ethical approval for the study was obtained from the National Health Research Ethics Committee (NHREC) of Nigeria-protocol number NHREC/01/01/2007-10/04/2017. The purpose of the research was clearly explained to the Nurses/Midwives or Health Attendants and a written consent was formally sought and obtained before the data collection commenced. All identifiers for the PHCs are removed from this study.

## Results

The aggregated results for the different components of the required physical infrastructure, medical equipment, essential drugs and personnel are presented in (Table 1, Table 1 (suite), Table 1 (suite 1)). The distribution of specific type of staff/personnel by availability in the PHCs is presented in (Table 2) and essential drugs are presented in (Table 3, Table 3 (suite), Table 3 (suite 1)). Details of the specific physical items and medical equipment are available on request.

**Table 1:** availability of essential physical infrastructure, equipment

Required Item	Number of items available N (%)	Number of PHCs
**Building and Premises**	N=12	(n=12)
	2 (16.7)	2
	3 (25.0)	2
	4 (33.3)	2
	5 (41.7)	1
	6 (50.0)	3
	7 (58.3)	1
	9 (75.0)	1
**Mean**	4.8 sd 2.1 (40 sd 17.5)	
**Median**	4.5 iqr 3 (37.5 iqr 25.0)	
**Rooms and spaces**	N=14	(n=12)
	2 (14.3)	1
	3 (21.4)	2
	4 (28.6)	4
	5 (35.7)	1
	6 (42.9)	2
	8 (57.1)	1
	9 (64.3)	1
**Mean**	4.8 sd 2.1 (34.3 sd 15.0)	
**Median**	4.0 iqr 2.5 (28.6 iqr 17.9)	
**Medical Equipment in the female ward**	N=35	(n=7)
	7 (20.0)	1
	9 (25.7)	1
	12 (34.3)	1
	17 (48.6)	1
	22 (62.9)	1
	23 (65.7)	1
	29 (82.9)	1
**Mean**	17 sd 8.1 (48.6 sd 23.1)	
**Median**	17 iqr 14 (48.6 iqr 40.0)	
**Ward for infant and child**	N=23	(n=0)
**welfare**	0	0

Note: SD - Standard Deviation; IQR - Interquartile range

**Table 1(suite):** availability of essential physical infrastructure, equipment

Required Item	Number of items available N (%)	Number of PHCs
**Medical equipment in the Labour room**	N=53	(n=12)
	15 (28.3)	1
	17 (32.1)	3
	18 (34.0)	1
	21 (39.6)	2
	22 (41.5)	2
	29 (54.7)	1
	30 (56.6)	1
	32 (60.4)	1
Mean	21.8 sd 5.7 (41.1 sd 10.8)	
Median	21 iqr 8.5 (39.6 iqr 16.0)	
**Medical equipment in the first stage room**	N=35	(n=1)
	15(42.9)	1
**Antenatal/Interview room**	N=25	(n=5)
	5 (20.0)	1
	6 (24.0)	2
	10 (40.0)	1
	12 (48.0)	1
Mean	7.8 sd 3.0 (31.2 sd 12.0)	
Median	6 iqr 4 (24.0 iqr 16.0)	
**Medical equipment in Laboratory**	N=29	(n=1)
	1	1
**Separate cleaner room**	N=15	(n=0)
	0	0
**Medical equipment in consulting cubicle**	N=19	(n=4)
	8 (42.1)	2
	9 (47.4)	1
	13 (68.4)	1
Mean	9.5 sd 2.4 (50.0 sd 12.6)	
Median	8.5 iqr 3 (44.7iqr 15.8)	

Note: **SD**- Standard Deviation; **IQR** -Interquartile range

**Table 1(suite 1):** availability of essential physical infrastructure, equipment

Required Item	Number of items available N (%)	Number of PHCs
**Staff room**	N=8	(n=1)
	5 (62.5)	1
**Record room**	N=7	(n=2)
	2 (28.6)	1
	3 (42.9)	1
**Other general Equipment**	N=7	(n=1)
	1(14.3)	1
**Staff/personnel (N=24)**	2 (8.3)	1
	3 (12.5)	2
	4 (16.7)	4
	5 (20.8)	3
	6 (25.0)	2
**Mean**	4.3 SD 1.2 (17.9 SD 5.0)	
**Median**	4 iqr 1.5 (16.7 iqr 6.3)	
	0 (0.0)	1
**Essential Medicine (N=93)**	4 (4.3)	1
	9 (9.7)	1
	11 (11.8)	1
	14 (15.1)	2
	18 (19.4)	1
	20 (21.5)	2
	23 (24.7)	1
	37 (39.8)	1
	51 (54.8)	1
**Mean**	18.4 SD 14.0 (19.8 SD 15.1)	
**Median**	16 iqr 11.5 (17.2 iqr 12.4)	


Note: **SD**: Standard Deviation; **IQR**: Interquartile range; N refers to the NPHCDA minimum standard number

**Table 2:** minimum standard for PHC in Nigeria: staff/personnel

S/N	Staff/personnel (N)	Number of PHC (n=12)	
		Available (Adequacy)	Not Available
1	Medical officer (1)	6	6
2	CHO (1)	5	7
3	Nurse/midwife (4)	8(0)	4
4	CHEW (3)	8(0)	4
5	Pharmacy technician (1)	1	11
6	JCHEW (6)	2(0)	10
7	Environmental Officer (1)	0	12
8	Medical records officer (1)	0	12
9	Laboratory technician (1)	1	11
10	Health Attendant/Assistant (2)	11(0)	1
11	Security personnel (2)	6 (0)	6
12	General maintenance staff (1)	2	10
Note: N refers to the NPHCDA minimum number of staff/personnel			

**Table 3:** minimum standard for PHC in Nigeria: essential drug list

		Number of PHC (n=12)	
S/N	Drug	Available	Not Available
1	Lidocaine - Topical,	2	10
2	Acetylsalicylic Acid - Tablet	0	12
3	Paracetamol - Oral liquid, tablet	8	4
4	Chlorphenamine - Oral liquid, tablet	1	11
5	Epinephrine (Adrenaline) - Injection	1	11
6	Promethazine - Tablet, oral liquid	5	7
7	Diazepam - Injection	4	8
8	Paraldehyde - Injection	0	12
9	Phenobarbital - Tablet	0	12
10	Atropine - Injection	0	12
11	Charcoal (activated) - Powder	0	12
12	Amoxicillin Capsule	6	6
13	Benzylpenicillin -Injection	3	9
14	Erythromycin - Tablet	1	11
15	Nitrofurantoin- Tablet	0	12
16	Streptomycin - Injection	1	11
17	Clofazimine -Capsule	0	12
18	Rifampicin - Capsule or tablet	1	11
19	Metronidazole -Tablet	4	8
20	Mebendazole -Tablet	7	5
21	Pyrantel - Oral liquid, tablet	4	8
22	Antifilarial	9	3
23	Benzoin - Compound	2	10
24	Tincture	2	10
25	Chlorhexidine -Solution	0	12
26	Iodine - Solution	6	6
27	Methylated spirit - Solution	8	4
28	Sodium hypochlorite - Solution	4	8
29	Benzoic salicylic acid (Whitfield's) - Ointment	1	11
30	Benzoyl peroxide - Cream or gel	0	12
31	Benzyl benzoate- Emulsion	1	11
32	Calamine -Lotion	2	10
33	Gentamicin -Ointment	2	10
34	Methyl salicylate - Ointment	1	11
35	Neomycin Bacitracin - Ointment, powder	1	11
36	Nystatin - Ointment, cream	1	11

**Table 3(suite):** minimum standard for PHC in Nigeria: essential drug list

		Number of PHC (n=12)	
S/N	Drug	Available	Not Available
37	Zinc oxide -Ointment	0	12
38	Ferrous salts - Oral liquid, tablet	3	9
39	Folic acid - Tablet	6	6
40	Tuberculin - Injection, PPD	1	11
41	Absorbent gauze bandages	5	7
42	Cotton wool (absorbent)	8	4
43	Disposable gloves,	9	3
44	Disposable syringes - 5 mL with needles(19, 21 Gauge)	6	6
45	Disposable syringes - 2 mL with needles(19, 21 Gauge)	6	6
46	Chloramphenicol - Ear drops	4	8
47	Artesunate - Suppositories	4	8
48	Quinine - Injection	3	9
49	Pyrimethamine + sulfadoxine - Tablet, oral liquid	2	10
50	Benzathine Penicillin -Injection	1	11
51	Co-trimoxazole - Tablet, oral liquid	1	11
52	Gentamicin -Injection	7	5
53	Phenoxymethylpenicillin - Tablet	1	11
54	Tetracycline -Capsule	4	8
55	Antileprosy drugs	4	8
56	Dapsone -Tablet	0	12
57	Amoebicide	0	12
58	Anthelmintics	1	11
59	Praziquantel -Table	5	7
60	Diethylcarbamazine -Tablet	1	11
61	Artemether + lumefantrine - Oral liquid, tablet	5	7
62	Artesunate + amodiaquine - Tablet	2	10
63	Ethambutol - Tablet	0	12
64	Isoniazid - Tablet	0	12
65	Pyrazinamide -Tablet	0	12
66	Rifampicin - Capsule, tablet	0	12
67	Ascorbic Acid (vitamin C) -Tablet	4	8
68	Calcium gluconate - Injection	0	12
69	Calcium salts -Tablet	3	9
70	Folic acid - Tablet	3	9

**Table 3(suite 1):** minimum standard for PHC in Nigeria: essential drug list

		Number of PHC (n=12)	
S/N	Drug	Available	Not Available
71	Vitamin A- Capsule	1	11
72	Hydrocortisone + lidocaine - Suppository	0	12
73	Hyoscine N-butyl bromide Tablet	2	10
74	Magnesium Sulphate - Injection	1	11
75	Magnesium trisilicate - Compound tablet, oral liquid	2	10
76	Misoprostol - Tablets	2	10
77	Oral Rehydration Salts	3	9
78	Senna - Tablet	0	12
79	Zinc - Oral liquid, tablet	1	11
80	Barrier methods - Condoms with or without spermicide	4	8
81	Oral contraceptives -Tablet	4	8
82	Poliomyelitis vaccine - Oral liquid	0	12
83	Rabies immunoglobulin - Injection	0	12
84	Tetanus vaccine - Injection	3	9
85	Chloramphenicol - Eye drops, ointment	2	10
86	Chlortetracycline - Eye ointment	1	11
87	Oxytocin -	3	9
88	Ergometrine - Tablet, injection	3	9
89	Chlorpromazine - Injection	0	12
90	Beclomethasone - Inhaler	0	12
91	Salbutamol - Tablet, inhaler	1	11
92	Water for injection - Injection	1	11
93	Spatulas	0	12

**Buildings and premises:** the minimum standard in this section comprises 12 physical infrastructure including land area, the colour of the building, a clean water source from a motorized borehole and clear signpost visible from entry and exit points among others. There was no record to ascertain the size of the land area in 4 PHCs. None of the PHCs met the standard in all the other 11 items. In fact, the majority did not have most of the items except for walls and roof and sanitary waste collection point. A clean water source from a motorized borehole was available only in one PHC. Aggregating the responses (**Table 1, Table 1 (suite), Table 1 (suite 1)**), each PHC had at least two of the 12 items in the expected minimum standard but none had all the 12 items. Only one PHC met the minimum standard in 9 out of the 12 and two facilities had only 2 items available. The mean availability was 40% with a standard deviation of 17.5%.

**Sufficient rooms and space:** none of the 12 PHCs had all the 14 items in this segment. The best performance was in the availability of delivery rooms in 10 out of the 12 PHCs and maternity/lying-in section in 9 out of 12 PHCs. However, the delivery rooms were not adequate in any of the 10 PHCs as none had the expected 2 rooms. Adolescent health service room and laboratory were available in only one PHC, and none of the PHCs had a food demonstration area and kitchen. Similar to the physical structures, the aggregated response revealed that only 1 PHC had 9 (64.3%) items available while 7 of the PHCs had less than 30% of the required items.

**Medical equipment in female ward:** female ward was available in 7 out of the 12 PHCs. Thirty-five types of medical equipment are expected to be in the female ward. Out of this, the least available number of equipment was 7 (20%) in one PHC and the highest was 29 (82.9%) in one PHC. Three PHCs had between 63-66% of the expected 35 items whereas 4 PHCs did not have up to 50% of the expected equipment. The mean availability was 48.6% with a standard deviation of 23.1%. Items such as graduated medicine cup, drinking mug, and thermometer (oral) were not available in any of the PHCs. Other equipments such as bowls, stainless steel with stand, dressing trolley, forceps jar, hand breast pump, thermometer rectal and tongue depressor was available only in one PHC. There was no ward for infant and child welfare in any of the 12 PHCs.

**Medical equipment in labour room:** all the 12 PHCs had a labour room but most of the 53 medical equipment that should be in the labour room was not available in many of the PHCs. Of the 53 equipment that should be in a labour room, the least available number of equipment was 15 (28.3%) in one PHC and the highest was 32 (60.4%) in one PHC. The mean availability was 41% SD 10.8%. Only 3 PHCs had between 54-60% of the 53 items. However, bedpan (adult stainless steel), ceiling fan, delivery couch, fetal stethoscope (aluminium), gloves (disposable pack of 100 - pedal), instrument tray, kidney dish, weighting scale (baby), and drip stand were available in 10-11 of the PHCs. None of the PHCs had angle poised lamp, thermometer jar, and oro-pharyngeal airway (set of 8) (Table 1, Table 1 (suite), Table 1 (suite 1)).

**Medical equipment in first stage room:** first stage room was available in only one PHC. Fifteen (42.9%) out of the 35 expected medical equipment in the First Stage room were available in the PHC.

**Medical equipment in antenatal/interview room:** antenatal/interview room was available in 5 PHCs. The expected minimum number of medical equipment in this room was 25. The mean availability in the 5 PHCs was 7.8 (31.2%). None of the 5 PHCs had up to half of the expected 25 medical equipment in the antenatal/interview room. Three of the PHCs had less than 30% of the items. Items such as hammer (reflex), ANC gowns for patients, mackintosh sheet were not available in any of the 5 PHCs.

**Laboratory:** laboratory was available only in one of the 12 PHCs. Out of the 29 medical equipment that should be in the laboratory, only urine dipstick (multistix) was available in the PHC.

**Separate room for cleaners:** none of the PHCs had a separate room for cleaners.

**Medical equipment in consulting cubicle:** consulting cubicle was available in 4 out of the 12 PHCs. All the four PHCs had one instead of two consulting rooms as stated in the NPHCDA minimum standard. Nineteen types of equipment were expected in the consulting room. Two of the 4 PHCs had 8 (42.1%) of the 19 items whereas one PHC had 68.4% of the items. The mean availability of the 19 items in the four PHCs was 50% with a standard deviation of 12.6%.

**Staff room:** a staff room was seen in only one PHC with 5 of the 8 expected items being available.

**Record room:** there was no record room in 10 PHCs. In one PHC there were two items and in the second PHC, three items in the identified record room were available. **Other general equipment**: among the other general equipment (n=7) expected in a PHC, only one item (mobile phone or communication radio) was available in one PHC. Other items such as ambulance vehicle, computer, internet services were not available in any PHC.

**Staff/personnel:** the mean staff/personnel availability was 4.3 SD 1.2 indicating about 18% of the expected 24 personnel. The least number of staff/personnel was 2 (8.3%) in one PHC. A breakdown of staff/personnel by specific PHCs (Table not shown) revealed that the two staff in this one PHC was CHO and one health attendant. The highest number of staff was 6 (25%) in 2 PHCs. The distribution of the specific staff/personnel is presented in Table 2. A Medical Officer was available in one half of the 12 PHCs and CHOs were in 5 PHCs. There was no nurse/midwife in 4 PHCs, and in the 8 PHCs where there was a nurse/midwife, none had the adequate number. All the 8 PHCs had just one nurse/midwife. On the contrary, almost all the PHCs had a health attendant/assistant (11/12). CHEWs were available in 8 PHCs but not in the adequate number whereas Junior CHEWs were in only 2 PHCs and again inadequate in number (one instead of 6 per PHC). Pharmacy technician and laboratory technician were available in only one PHC. There was no Environmental Officer and Medical Records officer in any of the PHCs. One Security personnel instead of 2 was available in 6 PHCs and 2 PHCs had a General Maintenance staff (Table 2).

**Essential drug:** ninety-three essential drugs, dressing and medical devices are expected to be in a PHC in Nigeria. Assessment of the availability in 12 PHCs in rural Edo State (Table 1, Table 1 (suite), Table 1 (suite 1)) shows that one PHC had none of the 93 indicated drugs; another two PHCs had less than 10% of the 93 essential drugs. Eight out of the 12 PHCs had between 11.8-39.8% of the drugs available and only one had more than half (54.8%) available. The mean availability was 19.8% SD 15.1%. In (Table 3,Table 3 (suite), Table 3 (suite 1)) a distribution of the specific drugs by their availability in the PHCs is presented. Twenty-four (25.8%) of the essential drugs such as acetylsalicylic acid tablet, paraldehyde injection, Phenobarbital tablet, atropine injection, and nitrofurantoin tablet were not available in any PHC. Twenty-two others (23.7%) were available only in one PHC (Table 3, Table 3 (suite), Table 3 (suite 1)).

## Discussion

The study was designed to investigate the availability of physical infrastructures, medical equipment, drugs and personnel for maternal and child health care in rural PHCs in a State in Nigeria. The study was part of an implementation project aimed at increasing the access of rural women to skilled pregnancy care. As the project was implemented in collaboration and cooperation with policymakers, health workers and community leaders, we hoped to provide substantive evidence of the nature of facility deficits in PHCs in the area that was pointed out in previous qualitative research conducted in the study area [6,19,20]. Through the translation of the research findings and partnership with relevant policy and community stakeholders, the idea was to build consensus and impetus for addressing the deficits in ways to improve the quality of facilities and infrastructures for the provision of maternal and child health care in the project communities. This, hopefully, would lead to improved facility utilization, and increase women´s access to skilled pregnancy care.

In order to justify our approach and increase stakeholders´ understanding of the process, we compared the infrastructures and medical facilities available in the PHCs with the benchmarks and standards that had been recommended by the NPHCDA in Nigeria. The NPHCDA is the national umbrella organization that constructs and provides policy oversight to all PHCs in the country [14]. However, for increased effectiveness in a country with a large and widely dispersed population, the management of the PHCs has been decentralized to State Primary Health Care Development Agencies (SPHCDAs) working in 36 States, and under the direct cooperation of 774 Local Government Councils in the country [14,26].

The results of our assessment of the facilities revealed significant shortfalls in all key infrastructures and medical equipment in the PHCs as compared to the national NPHCDA standards. The shortfalls and deficits were demonstrable in all areas assessed. These included buildings and premises, rooms and spaces in the PHCs, medical equipment in the antenatal and delivery rooms, laboratory facilities, and other related items. For example, while the NPHCDA standard provides for items in the buildings and premises category, the rate of availability in the PHCs was only 40 per cent. Also, while 10 of the 12 PHCs had delivery rooms, and 9 had lying-in rooms, the availability of medical items in the available delivery rooms was about 41 per cent. For an indicator such as delivery that manages the survival of pregnant women, the absence of equipment and facilities in that domain is worrisome. Specifically, key items such as bowls, stainless steel, dressing trolley, forceps jar, hand-held breast pump, thermometers, and tongue depressors could be found in only one of the 12 PHCs. It was also noteworthy that none of the PHCs had ambulances for the transfer of pregnant women experiencing complications of labour, mobile phones, internet or computers. Indeed, only one PHC had an official mobile phone for communication. In these days where strategic communication has taken frontline pre-eminence in the delivery of healthcare, the absence of these essential elements should worry the managers of PHCs in Nigeria. These results are consistent with studies that have observed low availability of personnel and essential medicines and equipment in primary and secondary health care facilities in Nigeria and other sub-Saharan African countries [17,21,27-30].

The results of this study have implications for the design of programs and processes for improving the delivery of maternal and child health care within PHCs in Nigeria. While the government has agreed to provide one per cent of its consolidated revenues for revamping primary health care in the country [26], it is important and critical that the funds are used wisely and effectively. We propose that the funds be used in an evidence-based manner, specifically to identify the deficits that exist through a purposefully designed needs assessment, and then to quantify the financial outlays needed to rectify the identified deficits.

The results of this study provide a framework for exploring this approach. In our next phase of research, we will quantify the financial resources needed to bring the facilities and equipment in these PHCs to the standards of the NPHCDA. We will then extrapolate the results to all PHCs in the two LGAs and to all PHCs in Edo State more generally. Such costs calculated for one of the 36 States of the country can be scaled up to other States, which will enable the determination of the costs for PHC recovery for the entire country. Without such a systematic approach, it will be difficult to achieve good results and effective outcomes for the current efforts being made by the federal government to rejig PHC delivery in the country.

This study has some limitations and also strengths. The major limitation is the sub-national scope of the study, without efforts made to cover the entire country. However, we took a random sample of PHCs in two rural LGAs in Edo State, with the aim of identifying the bottlenecks that exist specifically in rural PHCs, especially in communities where PHCs are the only source of maternal and child health care. The major strength of the study is that it was conducted with translational and interventional outcomes as the foci of attention. With the multi-disciplinary approach and the involvement of multiple stakeholders used as part of the methods, it is more likely that the results will find use in policy and program implementation. Our research approach that consisted of both interviews and verification of all the items also helped to increase data credibility.

## Conclusion

We conclude that PHCs in Etsako East and Esan South East Local Government Areas in rural Edo State of Nigeria have severe deficits in infrastructural facilities and medical equipment for the delivery of maternal and child health care. Efforts to improve these facilities will help improve the quality of delivery of maternal and child health and therefore reduce maternal and child mortality in the country.

### What is known about this topic

- Previous scholarly assessment of PHCs in Nigeria were focused on essential drugs and medical equipment with no attention to other indicators of service performance;
- Most of the studies were not specific to rural locations; were based on self-reporting of the indicators, and not supported by site verification.

### What this study adds

- The results of our assessment of the facilities revealed significant shortfalls in buildings and premises, rooms and spaces in the PHCs, medical equipment in the antenatal and delivery rooms, laboratory facilities, essential drugs, personnel and other related items;
- Key items such as bowls, stainless steel, dressing trolley, forceps jar, hand-held breast pump, thermometers, and tongue depressors could be found in only one of the 12 PHCs;
- None of the PHCs had ambulances for the transfer of pregnant women experiencing complications of labour or a computer, and only one PHC had an official mobile phone for communication.
